# Recent Advances in Production of Ecofriendly Polylactide (PLA)–Calcium Sulfate (Anhydrite II) Composites: From the Evidence of Filler Stability to the Effects of PLA Matrix and Filling on Key Properties

**DOI:** 10.3390/polym14122360

**Published:** 2022-06-10

**Authors:** Marius Murariu, Yoann Paint, Oltea Murariu, Fouad Laoutid, Philippe Dubois

**Affiliations:** 1 Laboratory of Polymeric and Composite Materials, Materia Nova Materials R&D Center & UMons Innovation Center, 7000 Mons, Belgium; yoann.paint@materianova.be (Y.P.); oltea.murariu@materianova.be (O.M.); fouad.laoutid@materianova.be (F.L.); 2 Laboratory of Polymeric and Composite Materials, Center of Innovation and Research in Materials and Polymers (CIRMAP), University of Mons (UMons), 7000 Mons, Belgium

**Keywords:** poly(lactic acid), PLA, biocomposites, mineral filler, calcium sulfate, natural gypsum, anhydrite II, melt–mixing, thermal and mechanical properties, crystallization, Vicat softening temperature, injection molding and extrusion, technical applications

## Abstract

The melt–mixing of polylactide (PLA) with micro- and/or nanofillers is a key method used to obtain specific end-use characteristics and improvements of properties. So-called “insoluble” CaSO_4_ (CS) β-anhydrite II (AII) is a mineral filler recently considered for the industry of polymer composites. First, the study proves that AII made from natural gypsum by a specifically thermal treatment is highly stable compared to other CS forms. Then, PLAs of different isomer purity and molecular weights (for injection molding (IM) and extrusion), have been used to produce “green” composites filled with 20–40 wt.% AII. The composites show good thermal and mechanical properties, accounting for the excellent filler dispersion and stability. The stiffness of composites increases with the amount of filler, whereas their tensile strength is found to be dependent on PLA molecular weights. Interestingly, the impact resistance is improved by adding 20% AII into all investigated PLAs. Due to advanced kinetics of crystallization ascribed to the effects of AII and use of a PLA grade of high L-lactic acid isomer purity, the composites show after IM an impressive degree of crystallinity (DC), i.e., as high as 50%, while their Vicat softening temperature is remarkably increased to 160 °C, which are thermal properties of great interest for applications requiring elevated rigidity and heat resistance.

## 1. Introduction

The high interest and progress in the production of biosourced polymers such as polylactide or poly(lactic acid) (PLA), is connected to a large number of factors, including the increase in requests for more environmentally sustainable products, the development of new biobased feedstocks and larger consideration of the techniques of recycling, increase in restrictions for the use of polymers with high “carbon footprint” of petrochemical origin, particularly in applications such as packaging, automotive, electrical and electronics industry, and so on [1,2,3,4,5,6,7,8].

Nowadays, when looking for a sustainable society and environmentally friendly products, the market turns to more “durable” applications, therefore important demands can be expected for new biomaterials which clearly offer multiple benefits to customers. Still, for many applications, the carbon footprint of products can be reduced by replacing “fossil carbon” with “renewable carbon” [9].

PLA, a biodegradable polyester produced from renewable resources, is one of the key bioplastics with the largest market significance due to its properties (high tensile strength and rigidity, good flexural strength, optical transparency) [2]. Due to its very interesting properties, PLA is currently receiving considerable attention for traditional applications such as packaging [10], as well as the production of textile fibers [11,12], and it also finds higher added value for durable/technical [9,13] and biomedical applications [14].

Regarding the use of PLA in durable applications, unfortunately, its application is somewhat limited because this biopolyester suffers from some shortcomings, e.g., poor thermal resistance, low heat distortion temperature and rate of crystallization, as well as rather high sensitivity to hydrolysis, whereas specific end-use properties are required. Therefore, at this time, an impressive number of studies concerning the production of novel PLA (nano)composites characterized by improved characteristics, such as better processability, enhanced mechanical properties and thermal resistance, flame retardancy, tailored electrical properties, longer durability to allow PLA utilization in applications requiring higher added value, have been undertaken. To reach the end-user demands, the properties of PLA can be tuned up by combining the polyester matrix with different dispersed phases: micro- and nano-fillers, reinforcing fibers, impact modifiers, plasticizers, other polymers, and various types of additives. Following different objectives, PLA has already been melt–mixed with CaCO_3_ [8,15], talc [16,17], kaolin [18], BaSO_4_ [19], and other mineral fillers, typically used in the industry of polymer composites.

It is worth mentioning that composites of PLA with CaSO_4_ (CS), as anhydrite or hydrated forms, have been primarily used in the field of biomedical applications for bone reparation and production of implant materials [20,21,22,23]. CS is considered as an uncommon biocompatible material which is completely resorbed following its implantation [21]. Nevertheless, few studies were devoted to the utilization of synthetic CS whiskers to reinforce polyvinyl chloride (PVC) [24,25], polypropylene (PP) [26,27], and polycaprolactone (PCL) [28]. Furthermore, in response to the demands for enlarging PLA applications while reducing its production cost, it has been previously disclosed by us and our collaborators that PLA can be effectively melt–blended with adequately thermally treated synthetic gypsum [29,30,31,32], a by-product directly issued from the lactic acid (LA) production process [33,34,35]. PLA can be successfully melt–blended with previously calcinated gypsum at 500 °C, so called β-anhydrite II (AII), which is less sensitive to moisture [2,32]. From the perspective of “green chemistry”, as well as economics, CS was considered a logical filler choice for PLA cost reduction due to its availability as a waste stream from LA production [36]. Moreover, as for other mineral-filled polymers, the addition of a third component into PLA-AII compositions, i.e., plasticizers [30], impact modifiers [32,37], clays [38], flame retardants (FRs) [2], other additives, has been considered to obtain composites with specific end-use properties. For more information on this topic, we suggest a short insight on the case study presented in a review published by us concerning the production and properties PLA composites [2].

Nevertheless, nowadays, the producers of natural gypsum are currently looking for new markets by proposing CS derivatives, such as AII, for new applications of higher added value, e.g., in the industry of polymer composites, paints, coatings, etc. Regarding so-called “insoluble” anhydrite (CS AII), somewhat regrettably, this filler is less known by potential users. Therefore, the information regarding the utilization of stable CS (AII) for reinforcing polymers is very limited compared to other mineral fillers (talc, CaCO_3_, kaolin, etc.), whereas the nature of CS derivatives or the necessity of thermally treatments at high temperature to produce stable fillers was much less studied [27,39]. There is also a misunderstanding connected to the quick absorption of water or high sensitivity to moisture, which is specific to CS hemihydrate (CaSO_4_ 0.5H_2_O) and to “soluble” anhydrite (i.e., AIII). Consequently, this confusion is detrimental for the utilization of stable forms of CS (i.e., AII) in melt–blending applications with polymers requiring fillers characterized by high thermal stability and low absorption of water/moisture. Accordingly, it is necessary to find additional methods to prove the stability and added value of AII as filler. To the best of our knowledge, the potential of this filler has not been identified sufficiently, therefore, further prospects are required to reveal its beneficial effects for different purposes. 

On the other hand, the earlier studies realized by us and our collaborators [2] were mostly limited to a specific PLA from the first generation (i.e., an amorphous PLA matrix, not available commercially today) and to the use of synthetic gypsum by-product as obtained directly from the LA process. Today, various PLA grades are available, characterized by different molecular weights, L-lactic acid isomer purity, as well as the presence of special additives, paving the way for new possibilities in applications [2,3]. In addition, it is known that the choice of PLA matrix is of high importance when following different techniques in processing (injection molding (IM), extrusion, 3D printing, etc.), aspects less considered in the previous studies. Still, as already mentioned, PLA is often in an amorphous state after any processing step, such as extrusion or IM, showing limited or poor heat resistance (low heat distortion temperature (HDT)). In fact, this is a kind of ‘Achilles’ heel’, limiting PLA use in engineering/technical applications [40]. This parameter (i.e., the degree of crystallinity (DC)) is particularly essential to control the PLA degradation rate, thermal resistance, as well as mechanical, optical, and barrier properties. The adequate choice of PLA matrix, and the combination with a filler that can increase the crystallization rate of PLA, could open the way to better performing composites designed for engineering applications requiring resistance at high temperature. 

Based on the prior art, the main goal of this study is to present recent experimental results and advances regarding the properties of mineral-filled biocomposites produced with CS AII made from natural gypsum and using PLA matrices of different molecular weights and isomer purity, mainly intended for processing by IM or extrusion. This will allow determining that the adequate choice of the PLA matrix is of key importance from the perspective of the application. Moreover, because CS AII is less known as a performant filler, one additional goal is to increase the interest in its utilization by experimentally proving its stability under harder testing conditions, such as following mixing in water as slurry. Regarding the PLA-AII composites, the study is focused on the characterization of their morphology and evidence for enhancement and tuning of thermal and mechanical properties connected to the nature of PLA and amounts of filler. However, it reveals some unexpected performances for special compositions, i.e., a remarkable increase in both DC and Vicat softening temperature (VST). Due to their properties, these “green” composites are of potential interest for utilization in the biomedical sector (e.g., via 3D printing) as biodegradable/rigid packaging and in technical applications requiring rigidity, heat resistance, and dimensional stability.

## 2. Materials and Methods

### 2.1. Materials

Three distinct PLA grades were investigated in the frame of the experimental program to consider different applications of and techniques for processing:

1. PLA 4032D (supplier: NatureWorks LLC, Blair, NE, USA), is a PLA of high molecular weight and melt viscosity designed for the extrusion of films and the realization of PLA blends. It is characterized by low D-isomer content (1.4%) and a melting temperature (T_m_) in the range of 155 to 170 °C and is abbreviated as PLA1.
2. PLA2: PLA 3051D is an IM grade for realization of products requiring low HDT (supplier NatureWorks LLC) characterized by higher D-isomer content (i.e., 4.3%) and a T_m_ in the range of 150 to 165 °C, according to the technical sheet of the supplier.
3. PLA3: PLA Luminy L105 (supplied by Total Corbion PLA (actually, TotalEnergies Corbion), Gorinchem, The Netherlands) is characterized by high L-isomer purity (L-isomer ≥99%, and implicit by very low content of D-isomer, <1%) and T_m_ of ca. 175 °C. PLA3 is a high flow PLA for spinning and IM, allowing the production of items with thin walls.

Table 1 shows the rheological information (i.e., melt flow rate (MFR) values) and the results of molecular characterizations by gel permeation chromatography (GPC), also referred to as size-exclusion chromatography (SEC) obtained using Agilent 1200 Series GPC-SEC System (Agilent Technologies, Santa Clara, CA, USA) and chloroform (at 30 °C) as the solvent (M_w_ being the weight-average molar mass expressed in polystyrene equivalent, the dispersity being the M_w_/M_n_ ratio between the weight- and number-average molar masses).

CaSO_4_ β-anhydrite II (CS AII) delivered as “ToroWhite” filler was kindly supplied by Toro Gips S.L. (Spain). According to the information provided by supplier, these products are obtained from selected food and pharma grades of high purity natural gypsum. They are characterized by high whiteness/lightness (L*), AII being an alternative of choice as a white pigment (TiO_2_) extender. Color measurements performed in the CIELab mode (illuminate D65, 10°) with a SpectroDens Premium (TECHKON GmbH, Königstein, Germany) have evidenced the high lightness of AII, i.e., L* of 95.8. Samples of CS dihydrate were also obtained from the same supplier for specific comparative tests (vide infra).

**Figure 1:**
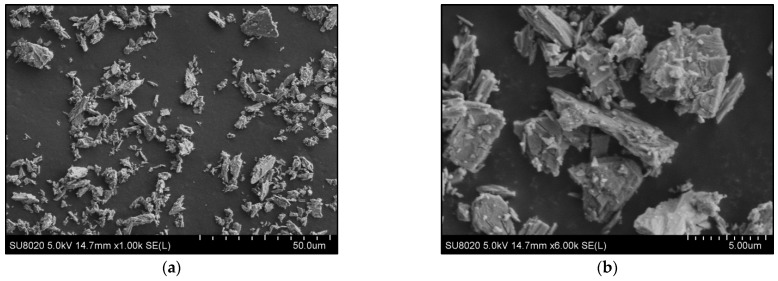
(**a**,**b**) SEM micrographs (SE mode) at different magnifications of CS AII microparticles.

Figure 1a,b show selected SEM pictures to illustrate the morphology of AII filler used in this study for melt-blending with PLAs. The granulometry of AII sample was characterized by Dynamic Light Scattering (DLS) using a Mastersizer 3000 laser particle size analyzer (Malvern Panalytical Ltd., Malvern, UK), the microparticles having a D_v50_ of 5.4 µm and a D_v90_ of 14.9 µm.

### 2.2. Specific Methods and Analyses to Demonstrate the Stability of AII as Filler

**Figure 2:**
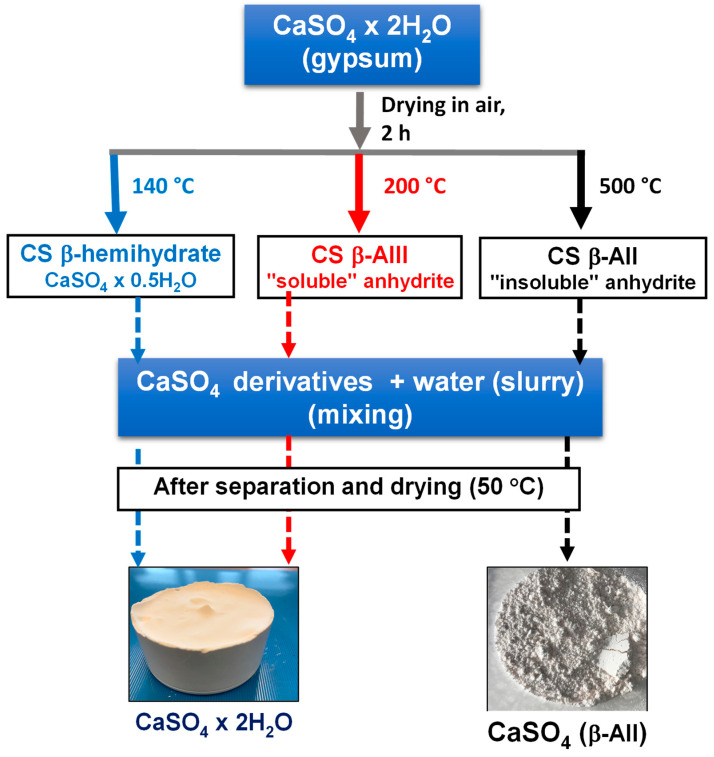
Experimental attempts to evidence the stability of CS β-AII by comparison to CS β-hemihydrate and CS β-AIII.

To evidence the distinct characteristics of AII, CS dihydrate was thermally treated during 2 h at different temperatures (140 °C, 200 °C, and 500 °C) in a Nabertherm B400 furnace (Nabertherm GmbH, Lilienthal, Germany) to obtain different forms of CS, respectively, CS hemihydrate, CS β-anhydrite III (AIII), and CS β-anhydrite AII (Figure 2). Then, the so-produced samples were characterized using TGA and XRD techniques. Furthermore, to test the stability of AII even after immersion in water, AII powders were mixed as a slurry (20%) in demineralized water for 24 h. The solid fraction (AII) was separated by sedimentation and centrifugation, maintained 24 h under a fume hood at room temperature (RT), and then dried under vacuum at low temperature (50 °C) for 2 h to remove the residual moisture. 

On the other hand, for sake of comparison, similar experiments were performed with CS (hemihydrate) and AIII, but these fillers were found to be extremely sensitive to water [41], leading to the formation of solid “blocky” structures of CS dihydrate (Figure 2).

### 2.3. Preparation of PLA-AII Composites

**Figure 3:**
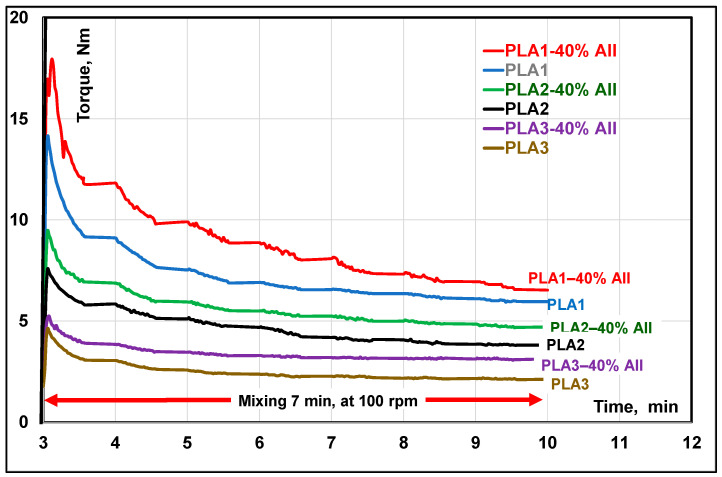
Comparative evolution of torque during melt-mixing of PLAs and PLA-AII composites using Brabender internal mixers.

All materials were carefully dried at 70 °C overnight to limit PLA degradation during processing at high temperature due to the presence of moisture. Starting from dry-mixed PLAs and CS (AII) blends, PLA composites were obtained by melt–compounding each of the three polyester matrices with 20% and 40 wt.% AII at 200 °C, using a Brabender bench scale kneader (Brabender GmbH &. Co. KG, Duisburg, Germany) equipped with “came” blades (conditions of processing: feeding at 30 rpm for 3 min, followed by 7 min melt–mixing at 100 rpm). The evolution of mechanical torque during the melt–mixing of PLAs and PLA−AII composites was followed and considered as primary rheological information (Figure 3).

In the subsequent step, the materials recovered after the melt–compounding process (after cooling in nitrogen liquid) were ground with a Pulverisette 19 (Fritsch GMBH, Idar-Oberstein, Germany), whereas the specimens for mechanical characterizations were obtained by IM, using a DSM micro injection molding (IM) machine (now Xplore, Sittard, The Netherlands), using the following processing conditions: temperature of IM = 200 °C, mold temperature = 70 °C. For the sake of comparison, neat PLAs were processed using similar conditions as with the mineral filled composites. Throughout this contribution, all percentages are given as weight percent (wt.%).

### 2.4. Methods of Characterization

(a) Thermogravimetric analyses (TGA) were performed using a TGA Q50 (TA Instruments, New Castle, DE, USA) by heating the samples under nitrogen or air from room temperature (RT) up to a max. 800 °C (platinum pans, heating ramp of 20 °C/min, 60 cm^3^/min gas flow rate).

(b) Differential Scanning Calorimetry (DSC) measurements were accomplished by using a DSC Q200 from TA Instruments (New Castle, DE, USA) under nitrogen flow. In the case of PLAs and PLA composites, the procedure was as follows: first heating scan at 10 °C/min from 0 °C up to 200 °C, isotherm at these temperature for 2 min, then cooling by 10 °C/min to −20 °C, and finally, a second heating scan from −20 to 200 °C at 10 °C/min. The first scan was used to erase the prior thermal history of the polymer samples. The events of interest linked to the crystallization of PLA during DSC cooling scan, i.e., the crystallization temperatures (T_c_) and the enthalpies of crystallization (ΔH_c_), were quantified using TA Instruments Universal Analysis 2000 software (Version 3.9A (TA Instruments—Waters LLC, New Castle, DE, USA)). Noteworthy, all data were normalized to the amounts of PLA from the samples. The thermal parameters were also evaluated in the second DSC heating scan and abbreviated as follows: glass transition temperature (T_g_), cold crystallization temperature (T_cc_), enthalpy of cold crystallization (ΔH_cc_), melting peak temperature (T_m_), melting enthalpy (ΔH_m_), and final DC (χ). The DC (degree of crystallinity) was determined using the following general equation:

$$\chi =\frac{(\Delta H_{m}\;-\;\Delta H_{cc})\;}{\Delta H_{m}^{0}\;\times \;W_{PLA}}\;\times \;100\;\left(\%\right)$$

where ΔH_m_ and ΔH_cc_ are the enthalpies of melting and of cold-crystallization, respectively, W is the weight fraction of PLA in composites, and $\Delta H_{m}^{0}$ is the melting enthalpy of 100% crystalline PLA considered 93 J/g [42]. Notable, the DC was calculated by subtracting the enthalpy of cold crystallization (∆H_cc_) and of pre-melt crystallization (if it was evidenced on DSC curves), from the enthalpy of melting (ΔH_m_).

To have information about the DC of specimens produced by IM, the properties of PLA and PLA-AII composites of interest were evaluated following the first DSC scan. The DSC technique was also used to evidence the transformation of gypsum by heating to 400 °C (the limit of instrument).

(c) Mechanical testing: Tensile tests were performed with a Lloyd LR 10K bench machine (Lloyd Instruments Ltd., Bognor Regis, West Sussex, UK) according to the ASTM D638-02a norm on specimens-type V at a crosshead speed of 1 mm/min. For the characterization of Izod impact resistance, a Ray-Ran 2500 pendulum impact tester and a Ray-Ran 1900 notching apparatus (Ray-Ran Test Equipment Ltd., Warwickshire, UK) were used according to ASTM D256 norm (method A, 3.46 m/s impact speed, 0.668 kg hammer). For both tensile and impact tests, the specimens produced by IM were previously conditioned for at least 48 h at 23 ± 2 °C under relative humidity of 50 ± 5%, and the values were averaged over minimum five measurements. 

(d) DMA (Dynamic Mechanical Analysis) were performed on rectangular specimens (60 × 12 × 2 mm^3^) obtained by IM (DSM micro-IM machine) using a DMA 2980 apparatus (TA Instruments, New Castle, DE, USA) in dual cantilever bending mode. The dynamic storage and loss moduli (E′ and E″, respectively) were determined at a constant frequency of 1 Hz and amplitude of 20 µm as a function of temperature from −20 °C to 140 °C, at a heating rate of 3 °C/min.

(e) Vicat softening temperature (VST) measurements were performed according to ASTM D1525, using HDT/Vicat 3-300 Allround A1 (ZwickRoell Gmbh & Co, Ulm, Germany) equipment. The samples with thickness of 3.2 mm were rectangular shaped (12 × 10 mm^2^). All samples were evaluated under a load of 1000 *g* and at a heating rate of 120 °C/h using minimum 3 specimens.

(f) Scanning Electron Microscopy (SEM) analyses on the PLA samples, previously cryofractured at a liquid nitrogen temperature, were performed using a Philips XL scanning electronic microscope (Eindhoven, Netherlands) at various accelerated voltages and magnitudes. For better information and easy interpretation, the SEM was equipped for both secondary (SE) and back scattered electrons (BSE) imaging. Reported microphotographs represent typical morphologies as observed at, at least, three distinct locations. SEM analyses of AII microparticles were performed at different magnifications in the SE mode (5 kV accelerated voltage). SEM analyses were also performed on the surfaces of selected specimens fractured by tensile or impact testing.

(g) X-ray diffraction (XRD) characterization: The morphological analysis of CS powders by X-ray diffraction was performed on a Siemens D5000 diffractometer (Siemens AG, Munich, Germany) using Cu Kα radiation (wavelength, 1.5406 Å) at RT, for 2θ from 10° to 90° (scanning step 0.026).

## 3. Results and Discussion

### 3.1. New Evidence of CS AII Stability as Filler for the Industry of Polymer Composites

**Figure 4:**
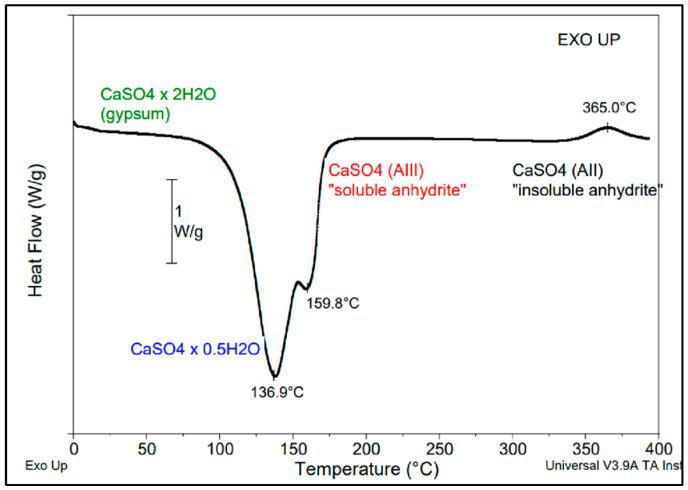
Thermal transformations of CS dihydrate as revealed by DSC heating to 400 °C (DSC method, 10 °C/min).

It is a noteworthy reminder that CS is available in several forms: dihydrate—CaSO_4_·2H_2_O (commonly known as gypsum), hemihydrate—CaSO_4_ 0.5H_2_O (Plaster of Paris, stucco, or bassanite), and different types of anhydrite [43,44,45]. The dehydration of gypsum (CS dihydrate) above 100 °C at low pressure (vacuum) or under air at atmospheric pressure, favors β-CS hemihydrate formation. An increase in the temperature to about 200 °C allows producing so called β-anhydrite III (β-AIII)—which is not stable, whereas calcination at temperatures higher than 350 °C (e.g., at 500–800 °C in an industrial process) allows obtaining stable β-anhydrite II (abbreviated as AII). The CS phases obtained by progressive dehydration and calcination of gypsum at higher temperatures are in the following order [43]: dihydrate → hemihydrate → anhydrite III → anhydrite II → anhydrite I (at temperatures higher than 1180 °C). DSC is a powerful tool of analysis that can be considered to evidence the thermal transformations of CS dihydrate during heating (Figure 4). Accordingly, in the first step, the gypsum was transformed at about 140 °C in β-CS hemihydrate. When the hemihydrate was heated at higher temperature it was converted into “soluble” anhydrite—AIII (endothermal process, a shoulder was observed at about 160 °C on DSC curve), and above 350 °C, “insoluble“ anhydrite (β-AII) was generated, as evidenced by the exothermal transformation on DSC curves.

To allow the use of CS in the production of polymer composites (e.g., based on polyesters, such as PLA), we restate that it is of prime importance to dry (dehydrate) the CS dihydrate or hemihydrate prior melt–compounding, or the use of stable anhydrite forms is required, keeping in mind the importance of minimizing free moisture. Indeed, PLA is stable in the molten state provided that it is adequately stabilized and dried to have a maximum acceptable water content of 250 ppm, or even below 50 ppm, in the case of processing at high temperature [3]. Moreover, following a comparative study, it has been reported elsewhere that synthetic β-AII (made from gypsum from the LA production process), is much better suited for melt–blending with PLA than β-AIII, which is by far too sensitive to atmospheric water absorption [29]. Indeed, AIII has a dramatically quick uptake of water, which was evidenced at the start of the thermogravimetric analyses, thus its rapid transformation to CS hydrated forms can be assumed. Moreover, in the case of AIII analyzed by XRD, it was reported that the high humidity triggered an instant transformation into CS hemihydrate (bassanite) [46]. Accordingly, due to its instability, AIII is not recommended for melt–blending with polymers with high sensitivity to degradation by hydrolysis during processing at high temperature.

**Figure 5:**
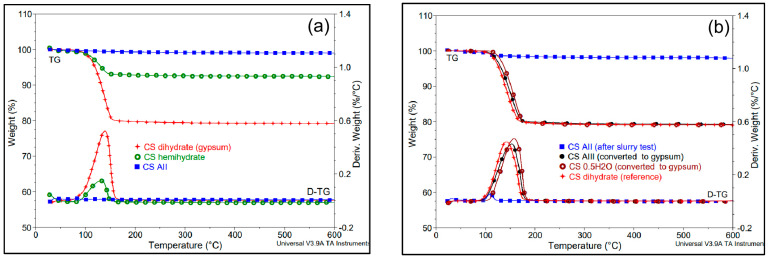
(**a**,**b**) Comparative TG and D-TG traces (20 °C/min, under N_2_). (**a**) CS hydrated forms (dihydrate and hemihydrate) and AII; (**b**) samples recovered after the slurry tests: CS (AII) after 24 h mixing in water as slurry; CS (AIII) converted to gypsum; CS hemihydrate converted to gypsum; CS dihydrate (reference).

Figure 5a shows the comparison of thermogravimetric analyses (TG) of CS derivatives produced from natural gypsum by thermal treatments at different temperatures. AII showed a particularly good thermal stability in all ranges of temperature (with a weight loss lower than 1% by heating to 600 °C). On the contrary, CS dihydrate and CS hemihydrate record a weight loss of 20–21% and 6–7%, respectively, in the dehydration process step (below 200 °C). 

The comparative TGAs of the recovered products after the slurry tests (Figure 5b) evidence only some low content of superficial water/moisture in the sample labelled “AII”, without fully excluding the presence of some traces of sub-hydrates. On the other hand, the total rehydration with the formation of gypsum in the case of CS hemihydrate and of AIII, was confirmed by the high amount of water lost during heating (21–22%).

**Figure 6:**
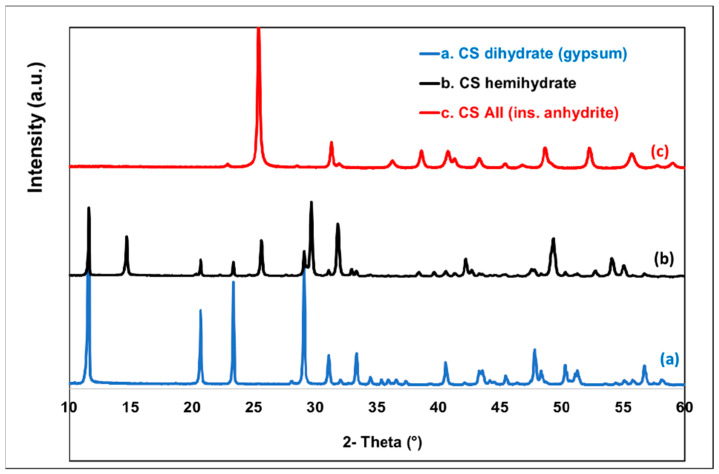
XRD patterns of CS AII and of CS hydrated forms.

Interestingly, the XRD technique is also largely used to evidence the differences between the various CS derivatives [46]. Figure 6 shows the comparative XRD patterns of products obtained following the transformation of gypsum at different calcination temperatures (see experimental section) to obtain β-CS hemihydrate and β-AII. AIII was not included here due to its sensitivity to moisture and its quick transformation into hydrated forms. For CS dihydrate (gypsum), five major diffraction peaks, i.e., (020), (021), (130), (041), and (−221), have been reported elsewhere [47]. Positions (2θ) of these peaks were confirmed in the present study, respectively, at 11.6°, 20.7°, 23.4°, 29.1°, and 31.1°, whereas additional XRD peaks were observed at higher 2θ angle. 

The diffractogram of β-CS hemihydrate featured specific peaks at 2θ ≈ 14.7, 25.6, 29.7, and 31.9° [48]. The presence of a supplementary peak at 2θ ≈ 11.6 (seen also for CS dihydrate) is reasonably ascribed to the inherent absorption of moisture, leading to traces of other CS hydrated forms. After the dehydration and thermal treatments (i.e., at 500 °C) of gypsum (monoclinic crystal system), the crystalline structure of the obtained CS β-anhydrite II (AII) was different, i.e., orthorhombic [49]. It was characterized by only one intense peak at 2θ ≈ 25.4° and a number of smaller ones at higher scattering angles [31].

**Figure 7:**
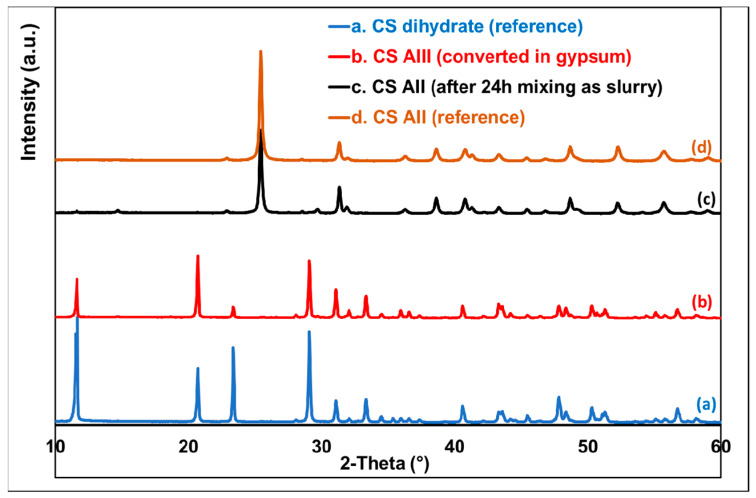
XRD patterns to evidence the stability of AII following the slurry tests.

On the other hand, Figure 7 shows the results of XRD analyses of selected samples recovered after the slurry tests. Accordingly, the following was found: when mixing in water only AII was stable, keeping its original crystalline structure as evidenced by the same XRD peaks, while the other CS forms (such as AIII and CS hemihydrate) were rehydrated to gypsum (CS dihydrate). 

These new results respond to the current questions asked by potential users requiring evidence of AII stability following contact with moisture/water. AII exhibited the closest packing of ions, which makes it highly dense and strong, whereas the absence of empty channels means it reacts slowly with water [43]. 

By considering its overall properties (high thermal stability, whiteness, low hardness (Mohs), very low solubility/rate of rehydration, others), AII can be considered a promising natural filler for the industry of polymer composites.

### 3.2. Characterization of PLA−AII Composites

First, it is important to point out that the results discussed hereinafter concern the use of AII without any surface treatments, whereas the PLAs used as polymer matrices are characterized by different molecular weights (M_WPLA1_ > M_WPLA2_ > M_WPLA3_) and rheology, to allow adapted melt processing techniques (e.g., extrusion or IM). Furthermore, for PLA2 and PLA3 (PLA grades of high fluidity designed for IM), the attention will be focused on the differences linked to the purity in L-lactic acid enantiomer.

By considering the evolution of torque values during melt mixing process as primary rheological information, in all cases the addition of filler (AII) into PLA led to the increase in mechanical torque/melt viscosity. Furthermore, the torque was clearly determined by the molecular weights of PLAs (see Figure 3, experimental part) and the following order was seen by melt–mixing at the temperature of 200 °C: PLA1–40% AII > PLA1 > PLA2–40% AII > PLA2 > PLA3–40% AII > PLA3.

#### 3.2.1. Morphology of PLA-AII Composites

After the grinding process, the microparticles of AII used for this study had a volume median diameter of ~5 µm (analysis of granulometry by DLS). The particulate filler was characterized by a low aspect ratio, whereas a shared morphology, i.e., particles with irregular shape and fibrillar/flaky aspect due to the cleavage of CS layers, was evidenced by SEM (Figure 1, section Materials). 

**Figure 8:**
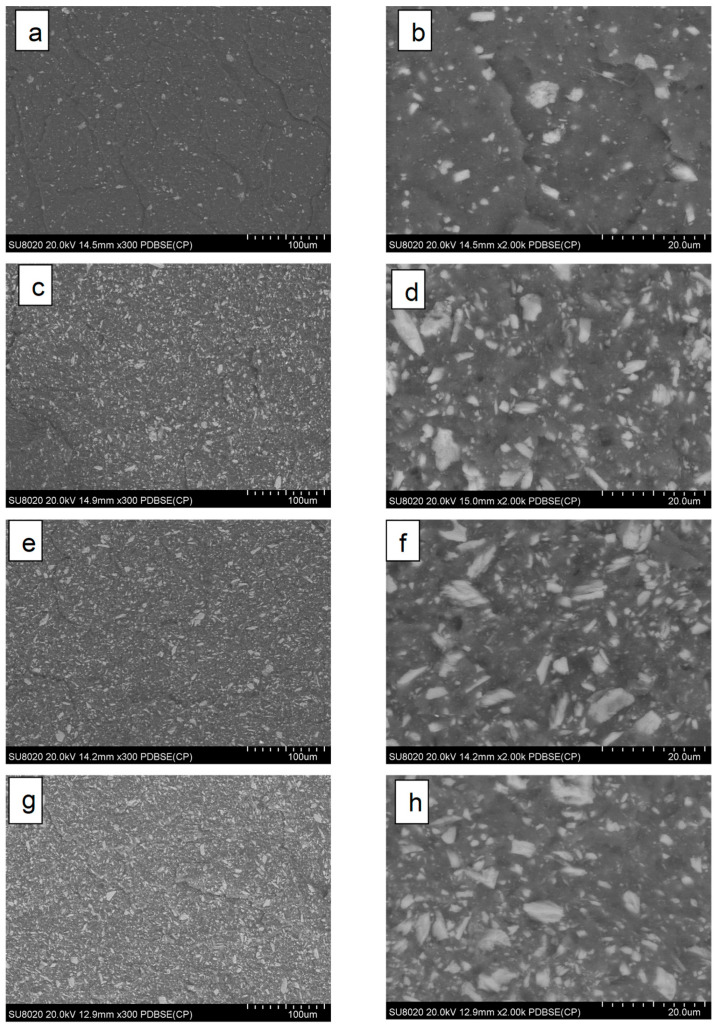
(**a**–**h**) SEM–BSE pictures at low and high magnification of cryofractured surfaces of PLA−AII composites having different PLA matrices: (**a**,**b**) PLA1−20% AII; (**c**,**d**) PLA1−40% AII; (**e**,**f**) PLA2−40% AII; (**g**,**h**) PLA3−40% AII.

Regarding the morphology of composites, for better evidence of filler distribution through PLA matrix, SEM imaging was performed using back scattered electrons (BSE) to obtain a higher phase contrast. Figure 8a–h shows representative SEM-BSE images of cryofractured surfaces of PLA−AII composites with 20–40% filler. 

Well-distributed/dispersed particles, with various geometries and quite broad size distribution were evidenced at the surface of cryofractured specimens. A cryofracture characterizing moderate, but effective adhesion between filler (AII) and PLA, can be assumed by considering the overall SEM images, but also considering the mechanical performances of composites. It is worth a reminder that such quality of dispersion was obtained without any previous surface treatment of filler. However, better individual particles dispersion was easily obtained at lower filler content (20% AII) (Figure 8a,b), whereas at high filling (40 wt.%), the presence of some aggregates/some zones with poorer dispersion was not totally excluded. Furthermore, it is difficult to conclude that following the melt–compounding with internal mixers led to important differences regarding the morphology of composites (i.e., in relation to the type of PLA matrix and the rheology of blends, which is essentially determined at similar amounts of filler by the molecular weights of PLA). 

#### 3.2.2. Thermogravimetric Analysis (TGA)

The results of thermal characterizations by TGA (Table 2) allow concluding that the addition of AII into different PLAs primarily leads to composites characterized by similar or better thermal properties than those of neat polymers processed under similar conditions. Interestingly, following the comparison of processed PLAs, PLA1 showed better thermal characteristics than PLA2 and PLA3. This difference was also seen in the case of composites, and it is reasonably ascribed to the higher molecular weights of PLA1. An increase in the onset of thermal degradation (T_5%_, temperature corresponding to 5% weight loss) and of maximum decomposition temperature (T_d_, from max. D-TG) was found as a general tendency by filling PLAs with up to 40% AII. However, more spectacular changes were observed when PLA2 or PLA3 was used as the polymer matrix. Furthermore, from the D-TG curves, it is observed that the rate of thermal degradation (wt.%/°C) at the temperature corresponding to the max. rate of degradation was much reduced/delayed in the case of composites, in quite good correlation with the amounts of filler. The enhancement of thermal stability by filling PLA with AII is a key-property in the perspective of processing and further application of such materials. For additional insight, the comparative TG and D-TG curves of neat PLAs and PLA−(20–40)% AII composites are shown in the Supplementary Material Figure S1a–c.

#### 3.2.3. Differential Scanning Calorimetry (DSC)

**Figure 9:**
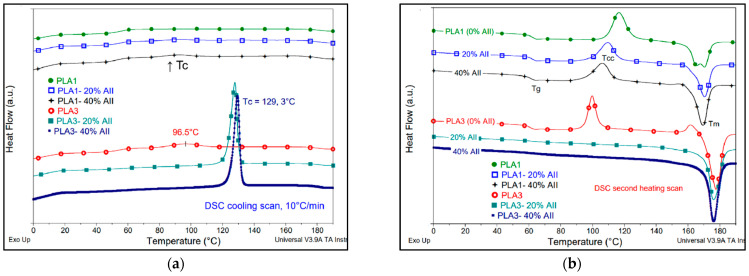
(**a**,**b**). Comparative DSC curves of neat PLAs (PLA1 and PLA3) and those of PLA1−AII and PLA3−AII composites obtained during (**a**) cooling and (**b**) second DSC heating (10 °C/min).

It is generally recognized that the PLAs of higher L-isomer purity (less than 1% D-isomer) are characterized by higher kinetics of crystallization, properties that can be improved in the presence of nucleating agents, to allow the utilization in applications requiring high HDT [50]. In contrast, PLA resins of higher D-isomer content (4–8%) are more suitable for thermoformed, extruded, and blow molded products, since they are more easily processed when the crystallinity is lower [3]. First, from the DSC analyses (Table 3 and Figure 9a,b) it was observed that the addition of AII had beneficial effects on the crystallization of PLA, distinctly evidenced for PLA1 and especially for PLA3 as a polymer matrix. 

Moreover, the DSC curves obtained during cooling and second heating scans clearly revealed that the association of AII with PLA3 of high L-isomer purity (≥99%) characterized by medium molecular weights (macromolecular chains with increased mobility during cooling process), yielded to composites characterized by surprising kinetics of crystallization and a high DC. In fact, the DC remarkably increased from 31% (neat-PLA3 processed) to about 60% in composites (PLA3−(20–40)% AII). Moreover, the effect of the filler was also significant using PLA1 as the matrix (PLA of higher molecular weights, D-isomer = 1.4%), the composites being characterized by better/moderate crystallization ability, determined by the level of filler: the DC of PLA1 (1.8%) increased in composites up to about 20%.

Still, using PLA2 with higher D-enantiomer content (4.3%), there were no important changes in crystallinity (Table 3 and Figure S2 from the Supplementary Material). The DC of neat PLA2 and of composites remained very low (DC < 2%) and only were slightly affected by the amount of filler. Regarding the cold crystallization process recorded in the second DSC scan, PLA2 had a lower crystallization ability (T_cc_ determined at high temperatures, i.e., 133–135 °C), whereas for PLA1 samples, T_cc_ decreased with the amount of filler, from 116 °C to 106 °C. However, by comparing the neat PLAs and their respective composites, for most of the samples, there was no significant modification of glass transition and melting temperatures (T_g_ and T_m_). 

In relation to the results of DSC characterizations, it was once more proved that the highest kinetics of crystallization/DC are obtained by reducing the molecular weights of PLA and using PLA of higher L-enantiomer purity [51], i.e., for PLA3. Still, the addition of AII into PLA3 leads to composites of interest for technical applications (by considering the overall performances of composites), because they show a superior DC, properties reasonably ascribed to the nucleating effects of filler and inherent characteristics of the polymer matrix.

#### 3.2.4. Mechanical Characterizations

**Figure 10:**
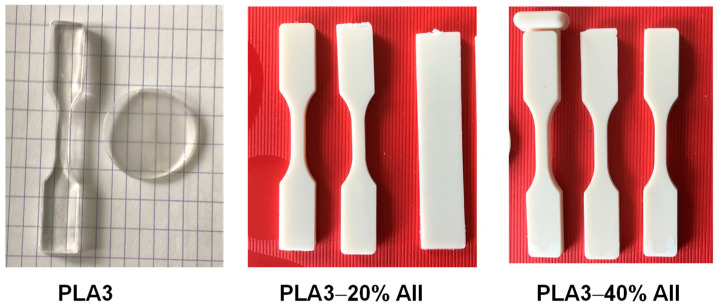
Selected pictures to illustrate the aspect of specimens of PLA and PLA−AII composites produced by IM.

The specimens for mechanical testing (Figure 10) were produced by IM (see experimental section). The strength of particulate-filled polymer composites depends, to a great extent, on the properties of the matrix, the interfacial adhesion between the matrix and dispersed phase, the filler shape, size, and amount [52]. Noteworthy, comprehensive studies regarding the interfacial adhesion between the PLA and microfiller (AII) have been realized using different techniques by the research group of Pukánszky B. and collab. [53].

**Figure 11:**
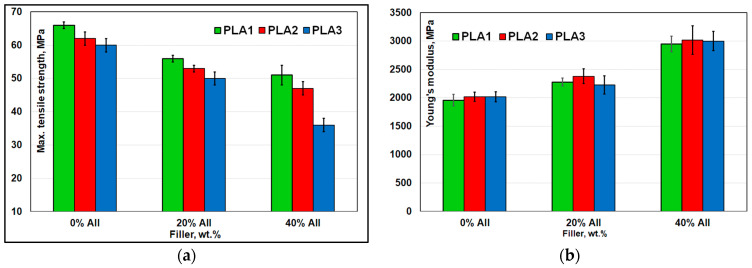

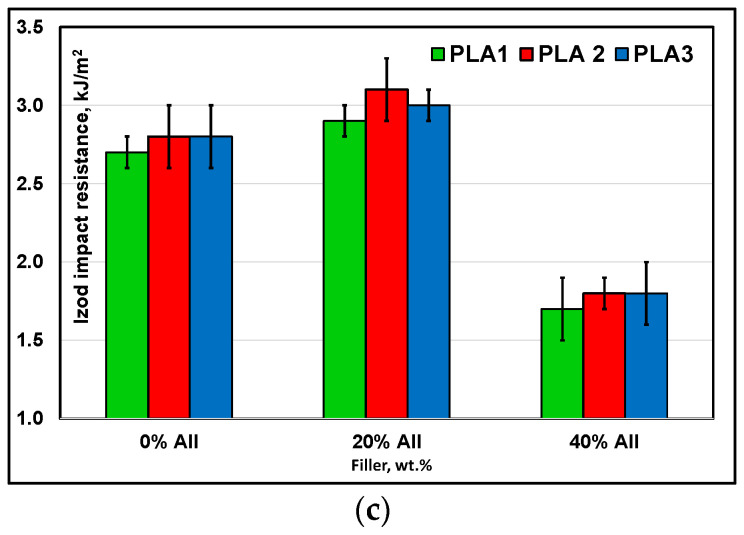
(**a**–**c**). Comparative mechanical characterizations of PLAs with different amounts of filler: (**a**) Maximum tensile strength; (**b**) Young’s modulus; (**c**) Izod impact resistance (notched specimens).

Figure 11a–c summarizes the results of mechanical characterizations of neat PLAs and their respective composites filled with 20–40% AII. For composites, the tensile strength gradually decreased with the loading of filler, e.g., adding 20% AII, from 60–66 MPa (neat PLAs) to 50–56 MPa for composites, values which are of real interest for engineering applications. In contrast, Young’s modulus (Figure 11b) was significantly enhanced (from ≈ 2000 MPa, neat PLAs) to a value of about 3000 MPa by filling with 40% AII, without any significant influence linked to the nature of PLAs. Moreover, when we compared the values of tensile strength (σ) of neat PLAs and PLA-AII composites, it was observed that they followed the same order (σ_PLA1_ > σ_PLA2_ > σ_PLA3_) as that of molecular weights (M_wPLA1_ > M_wPLA2_ > M_wPLA3_). Accordingly, at high filling (40% AII), it is worth noting the tensile strength of composites obtained using PLA1 and PLA2 as matrix (51 MPa and 47 MPa, respectively). As expected, all PLAs had low nominal strain at break (about 5%), values that decreased to 2–3% for composites.

Regarding the impact resistance, it has been reported in the literature that in some cases, well dispersed particulate fillers, at optimal loadings or having specific surface treatment, can contribute to the increase in impact properties [54,55]. Interestingly, the Izod impact resistance of composites (Figure 11c) was slightly improved by incorporating 20% AII in each of the three studied PLAs, whereas a further increase in filler content to 40% led to an important reduction in this parameter. In the case of PLA−20% AII composites, it is once more proved that well distributed/dispersed rigid/particulate microparticles can contribute to the dissipation of impact energy by reducing the crack propagation by different mechanisms (e.g., by crack-bridging [56] or crack pinning, which require certain adhesion between polymer and filler; whereas the debonding at matrix–particle interface is also for consideration as mechanism [48]). However, additional SEM images performed on fractured samples by tensile or impact testing (Supplementary Material, Figures S3 and S4) suggested that at the interface zone (PLA matrix–filler) were seen both regions, accounting for the good/moderate adhesion due to the maintaining of intimate physical contact between constituents after the mechanical solicitation (which explains the noticeable tensile properties), and zones of debonding or shear yielding, traditionally ascribed to a toughening mechanism with rigid particles, with contribution in reducing the cracking and in dissipating the energy of impact solicitation [37,54,55].

On the other hand, at higher filling (i.e., 40 wt.%), due to the inherent presence of more heterogeneous (mechanically weak) regions, e.g., aggregates of microparticles, they may act as stress concentrators, causing a decrease in tensile and impact resistance. Therefore, for applications in which the impact strength is a key-concern, these composites need to be modified to fulfil the industry requirements [2]. Modification of filler (AII) by special surface treatments or/and the addition of a third component into PLA–AII composites, i.e., a plasticizer [55], an impact modifier [37,57], etc., can represent alternatives of choice for better impact resistance performances.

#### 3.2.5. Dynamic Mechanical Analysis (DMA)

**Figure 12:**
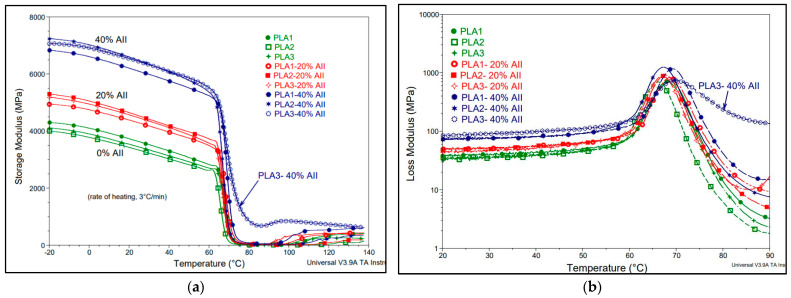
(**a**,**b**). Dependencies of (**a**) E′ (storage modulus) and (**b**) E″ (loss modulus) vs. temperature: neat PLAs compared with PLA−AII composites.

DMA has been used to provide information about the performances of PLA-AII composites in a broad temperature range (i.e., from −20 °C to 140 °C). Figure 12a,b shows the evolution of storage and loss modulus (E′ and E″, respectively) of neat PLAs and their composites as a function of the temperature. In correlation with AII percentage, E′ increased distinctly in the low-temperature glassy region for all composite samples (Figure 12a), trends that are like those recorded for the evolution of Young’s modulus in tensile tests, highlighting the reinforcing effect of filler. This increase is ascribed to considerable interfacial properties, allowing the stress transfer at low deformations, which finally is expressed in the enhancements of E′ and Young modulus with filler content [48]. Undeniably, as it is revealed as key example in Table 4, at the temperature of 60 °C (very close to T_g_), the loading of filler was responsible for the level of E′, apparently without some influence linked to the nature of PLA matrix. E′ was twofold increased for PLA composites filled with 40% AII compared to the unfilled PLAs.

Figure 12b shows the evolution of E″ (loss modulus) as a function of temperature. The results showed that the composites were characterized by slightly higher E”, determined by the filler loading, an increase assigned to the contribution of the mechanical loss generated in the interfacial regions [48]. However, the max. peak of E″ ascribed to the T_g_ zone was only slightly changed, from 66–68 °C for the neat PLAs to max. 70 °C upon AII filling, results that are in good agreement with DSC data. 

Still, due to the highly filling and increased crystallinity at higher temperatures (i.e., in the range 80–140 °C), PLA3-40% AII composites showed the most important enhancements of both, E’ and E”. As demonstrated under dynamic solicitation by DMA, melt–blending of PLA with AII offers the possibility to produce PLA composites for applications requiring enhanced mechanical rigidity and higher temperature of utilization.

#### 3.2.6. Vicat Softening Temperature (VST)

**Figure 13:**
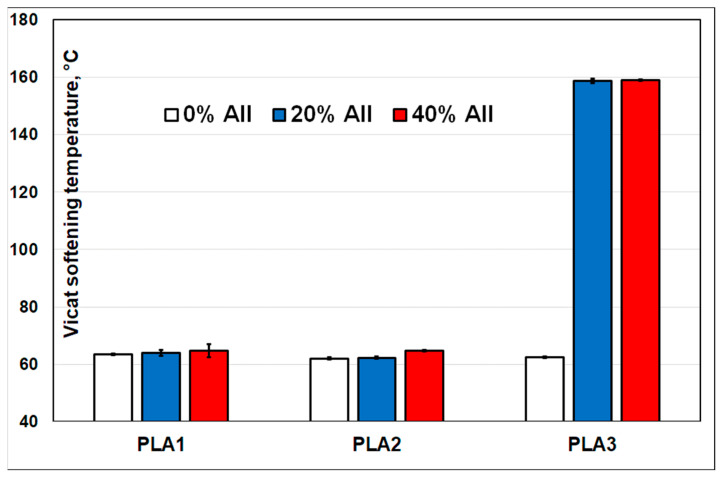
Vicat softening temperature (VST) of PLAs with different amounts of filler.

It is generally assumed that the fillers can be effective reinforcemnt phases leading to the improvement in specific thermal properties, such as HDT and VST, mostly in semi-crystalline polymers (polyethylene (PE), PP, polyamide (PA), etc.). The VSTs of neat PLAs were around 62–63 °C (Figure 13), whereas the addition of 20–40% filler into PLA1 and PLA2 led only to a slight increase in VST to about 65 °C. On the other hand, somewhat unexpectedly, when considering the melt processing conditions (the temperature of the mold was around 70 °C, without any additional annealing process to obtain higher crystallinity), the most remarkable enhancements were obtained for PLA3−AII composites, i.e., an increase in VST to 160 °C, primarily ascribed to the high level of crystallinity. In fact, the IM tests highlighted that the association PLA3 with AII leads to PLA composites characterized by remarkable crystallization kinetics, that allows the production of items with high DC. Indeed, the comparative DSC analyses performed on IM specimens (Supplementary Material, Figure S5a–c), confirmed the high DC (49–53%) of PLA3−AII composites, results that are in good agreement with those presented in the section thermal characterizations (DSC).

Before concluding, it is important to mention that the composites concerned in this study need further optimization by taking into account the constraints imposed by application (e.g., the amount of filler is a key-parameter), and this it is expected to be realized following their production in higher quantities using for melt–compounding twin-screw extruders. Moreover, in the frame of forthcoming contributions, it will be important to reconfirm the performances of composites using optimized processing conditions (extrusion, IM, etc.) and also to determine other characteristics of interest (flexural strength, HDT, rheological behaviour and MFI, evolution of molecular parameters as function of the residence time at high temperature and shear, etc.). Furthermore, the studies on crystallization mechanisms using alternative techniques of investigation (polarized light microscopy (POM), XRD, etc.), are of further concern.

## 4. Conclusions

The study provides answers to current requests regarding the production of environmentally friendly materials using PLAs as a matrix (biosourced and biodegradable) and the utilization of mineral fillers such as CS β-AII made from natural gypsum. 

New tests and specific characterizations were performed to prove the stability of AII as produced by the calcination of gypsum at high temperature (i.e., 500 °C), in comparison to other CS derivatives. Characterized by excellent thermal stability and low absorption of moisture/water of rehydration, so called “insoluble” AII is stable, maintaining its crystal structure even after mixing in water as slurry. Moreover, the overall results confirmed that CS β-AII is a filler of real interest for the industry of polymer composites. 

PLAs of different L-lactic acid isomer purity and molecular weights (as supplied for distinct processing techniques) were used to produce by melt–compounding composites filled with 20–40% AII. The addition of filler leads to composites characterized by enhanced thermal stability and increased rigidity (Young’s modulus), determined by the amount of filler. Interestingly, PLA impact resistance was not decreased when up to 20% filler was added, whereas the ultimate strength properties were dependent on the molecular characteristics of PLA matrix, keeping a similar order (PLA1 > PLA2 > PLA3). The values of tensile strength (e.g., 50–56 MPa, for PLA−20% AII composites) are of real interest for engineering applications. Melt–blending PLA with AII leads to two-fold enhancement of storage modulus (at 60 °C) and offers the possibility to use PLA in applications requiring enhanced mechanical rigidity and/or higher temperature of utilization. The good thermomechanical proprieties are ascribed to the fine distribution and dispersion of AII within PLA matrix, and favorable interfacial interactions between components. 

Advanced kinetics of crystallization linked to the addition of AII were evidenced by DSC for PLA3 of high L-isomer purity (≥99%) and lower molecular weights. Still, following the IM processing, the properties of crystallization remain impressive in the case of PLA3 filled with 20–40% AII (DC of about 50%), whereas a VST of 160 °C was attained on IM specimens (only 63 °C for the neat PLA). By considering the overall performances (tensile strength, stiffness, VST, other specific properties), these composites are proposed for development/production at larger scale, as a quick answer to current requests for the use of PLA-based products in engineering applications. Nevertheless, by the carefully choosing the PLA matrix, these “green” mineral filled (AII) composites can be designed for processing by IM, extrusion, thermoforming, and 3D printing. 

## Figures and Tables

**Table 1:** Characteristics of PLAs used as the polymer matrix.

PLA Matrix	M_w_	M_w_/M_n_ (Dispersity)	D-Isomer, %	MFR *, g/10 min
PLA1	209,000	2.0	1.4	7
PLA2	182,000	2.0	4.3	10–25
PLA3	133,000	1.9	<1.0	70

* Values indicated by supplier: Melt Flow Rate (MFR) measurements at 210 °C, 2.16 kg.

**Table 2:** Thermal parameters of PLAs and PLA-AII composites as determined by TGA.

Sample	Onset of Thermal Degradation (T_5%_), °C	Temp. at Max. Rate of Degradation, °C (From D-TG)	Max. Degradation Rate, wt.%/°C (From D-TG)
PLA1	341	376	2.7
PLA1−20% AII	345	382	2.2
PLA1−40% AII	342	378	1.7
PLA2	317	362	2.1
PLA2−20% AII	320	372	2.2
PLA2−40% AII	330	377	1.7
PLA3	320	369	2.5
PLA3−20% AII	330	370	2.1
PLA3−40% AII	335	378	1.5

**Table 3:** Comparative DSC results of neat PLAs and PLA–AII composites obtained using different PLA grades as polymer matrix (second DSC heating scan, by 10 °C/min).

Sample (%, by Weight)	T_g_ (°C)	T_cc_ (°C)	ΔH_cc_ (J g^−1^)	T_m_ * (°C)	ΔH_m_ (J g^−1^)	χ * %
PLA1	63	116	35.4	166; 170	37.1	1.8
PLA1−20% AII	62	109	26.2	170	37.8	12.5
PLA1−40% AII	61	106	27.4	169	45.4	19.4
PLA2	61	135	1.6	154	2.3	0.8
PLA2−20% AII	61	133	5.7	153	6.5	0.9
PLA2−40% AII	62	133	3.5	153	5.3	1.9
PLA3	61	100 (161)	28.9 (5.2)	177	62.7	30.8
PLA3−20% AII	ND **	-	-	176	55.7	59.9
PLA3−40% AII	ND	-	-	176	55.3	59.5

* χ-DC as calculated by subtracting ΔH_cc_ from ΔH_m_ and by considering an enthalpy of 93 J/g for 100% crystalline PLA; ND: ** not detectable on DSC curve.

**Table 4:** Effects of AII addition on the values of storage modulus (E′) at the temperature of 60 °C for different PLA matrices.

AII Content →	0%	20%	40%
↓ PLA Matrix	↓ Storage Modulus (E′), GPa		
PLA1	2.8	3.5	5.2
PLA2	2.6	3.7	5.4
PLA3	2.7	3.6	5.5

## Data Availability

Not applicable.

## Supplementary Materials

The following supporting information can be downloaded at: https://www.mdpi.com/article/10.3390/polym14122360/s1, as Supplementary Material: Figure S1a–c. TG and D-TG curves of neat PLAs and PLA−(20–40)% AII composites (under air, 20 °C/min): (a) PLA1 and PLA1−AII; (b) PLA2 and PLA2−AII; (c) PLA3 and PLA3−AII composites; Figure S2: Comparative DSC curves of neat PLA2 and those of PLA2−AII composites, as obtained during second DSC heating (10 °C/min).; Figure S3: SEM images (SE mode) of PLA−AII composites performed on the fractured surfaces obtained after tensile testing.; Figure S4: SEM images (SE mode) of PLA1−(20–40)% AII composites performed on fractured surfaces obtained by impact testing.; Figure S5a–c. Comparative DSC analyses on injection molded specimens of PLA and PLA−AII composites assessing for the higher degree of crystallinity (DC) of PLA3−AII composites (first DSC scan, 10 °C/min).
