# Supplementary material for: Recent Advances in Production of Ecofriendly Polylactide (PLA)–Calcium Sulfate (Anhydrite II) Composites: From the Evidence of Filler Stability to the Effects of PLA Matrix and Filling on Key Properties
Source: Polymers (Basel). 2022 Jun 10;14(12):2360. doi: 10.3390/polym14122360 (PMC9228668; doi:10.3390/polym14122360)
Supplement: Supplementary file 1 [file polymers-14-02360-s001.zip › polymers-1727760-supplementary.pdf]

polymers

MDPI

Supplementary Material

Recent Advances in Production of Ecofriendly Polylactide (PLA)–Calcium Sulfate (Anhydrite II) Composites: From the Evidence of Filler Stability to the Effects of PLA Matrix and Filling on Key Properties

Marius Murariu [1,*], Yoann Paint [1], Oltea Murariu [1], Fouad Laoutid [1] and Philippe Dubois [1,2,*]

1 Laboratory of Polymeric and Composite Materials, Materia Nova Materials R&D Center & UMons Innovation Center, 7000 Mons, Belgium; yoann.paint@materianova.be (Y.P.); oltea.murariu@materianova.be (O.M.); fouad.laoutid@materianova.be (F.L.).
2 Laboratory of Polymeric and Composite Materials, Center of Innovation and Research in Materials and Polymers (CIRMAP), University of Mons (UMons), 7000 Mons, Belgium
* Correspondence: marius.murariu@materianova.be (M.M.); philippe.dubois@umons.ac.be (P.D.); Tel.: +32-65-554976 (M.M.); +32-65-373000 (P.D.); Fax: +32-65-373054 (P.D.)

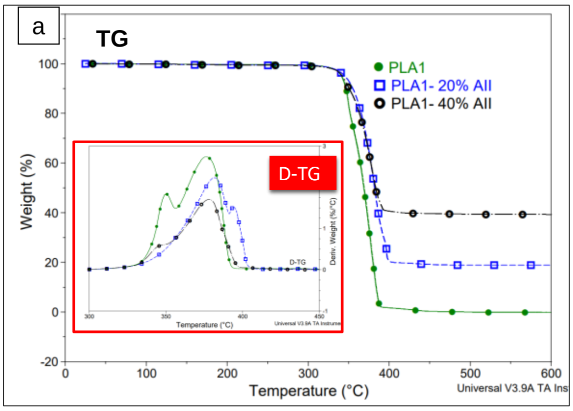

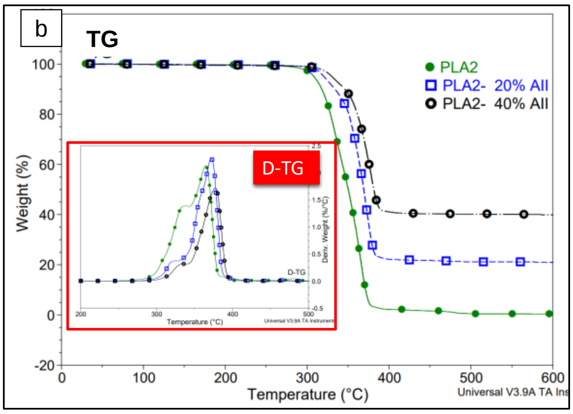

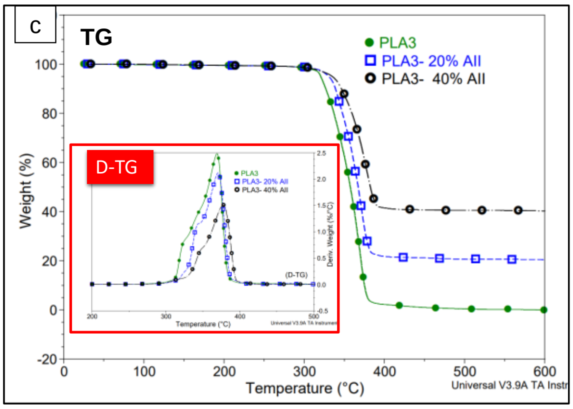

Figure S1 (a-c). TG and D-TG curves of neat PLAs and PLA–(20–40) % AII composites (under air, 20°C/min): (a) PLA1 and PLA1–AII; (b) PLA2 and PLA2–AII;

(c) PLA3 and PLA3–AII composites.

Comment: *The addition of AII into different PLAs is leading to composites characterized by similar or better thermal properties than those of neat polymers processed under similar conditions..*

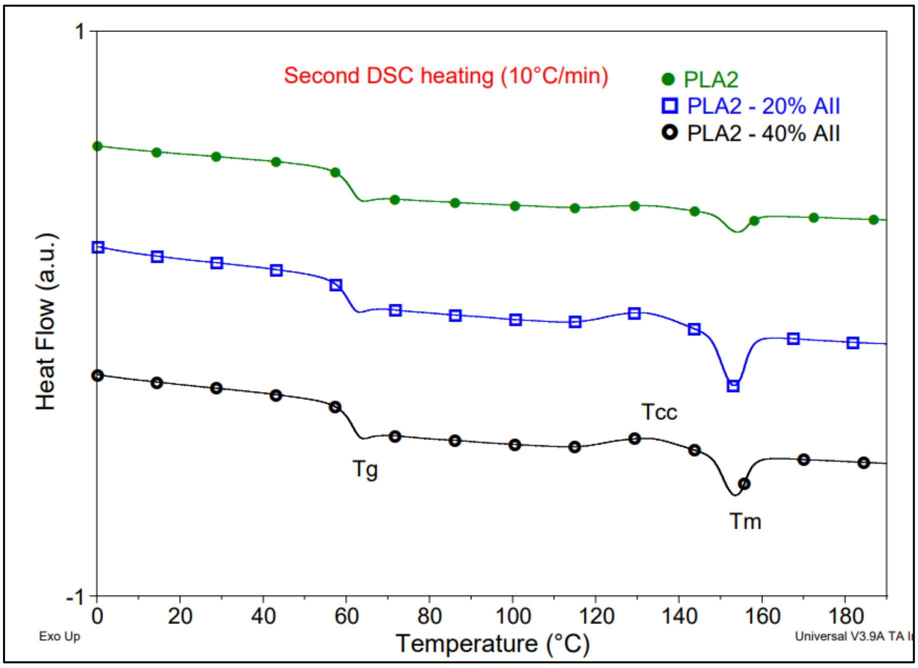

Figure S2. Comparative DSC curves of neat PLA2 and those of PLA2–AII composites as obtained during second DSC heating (10°C/min).

Comment: *Because PLA2 has high D-isomer content (4.3%), the degree of crystallinity (DC) of neat PLA2 and of composites, following the cooling by 10°C/min, is very low (DC < 2%) and only slightly affected by the amounts of filler (AII). Regarding the cold crystallization process seen in the second DSC scan, by comparison to the other PLAs considered in the study, PLA2 has the lowest crystallization ability (T_{cc} determined at high temperatures, i.e., 133-135°C).*

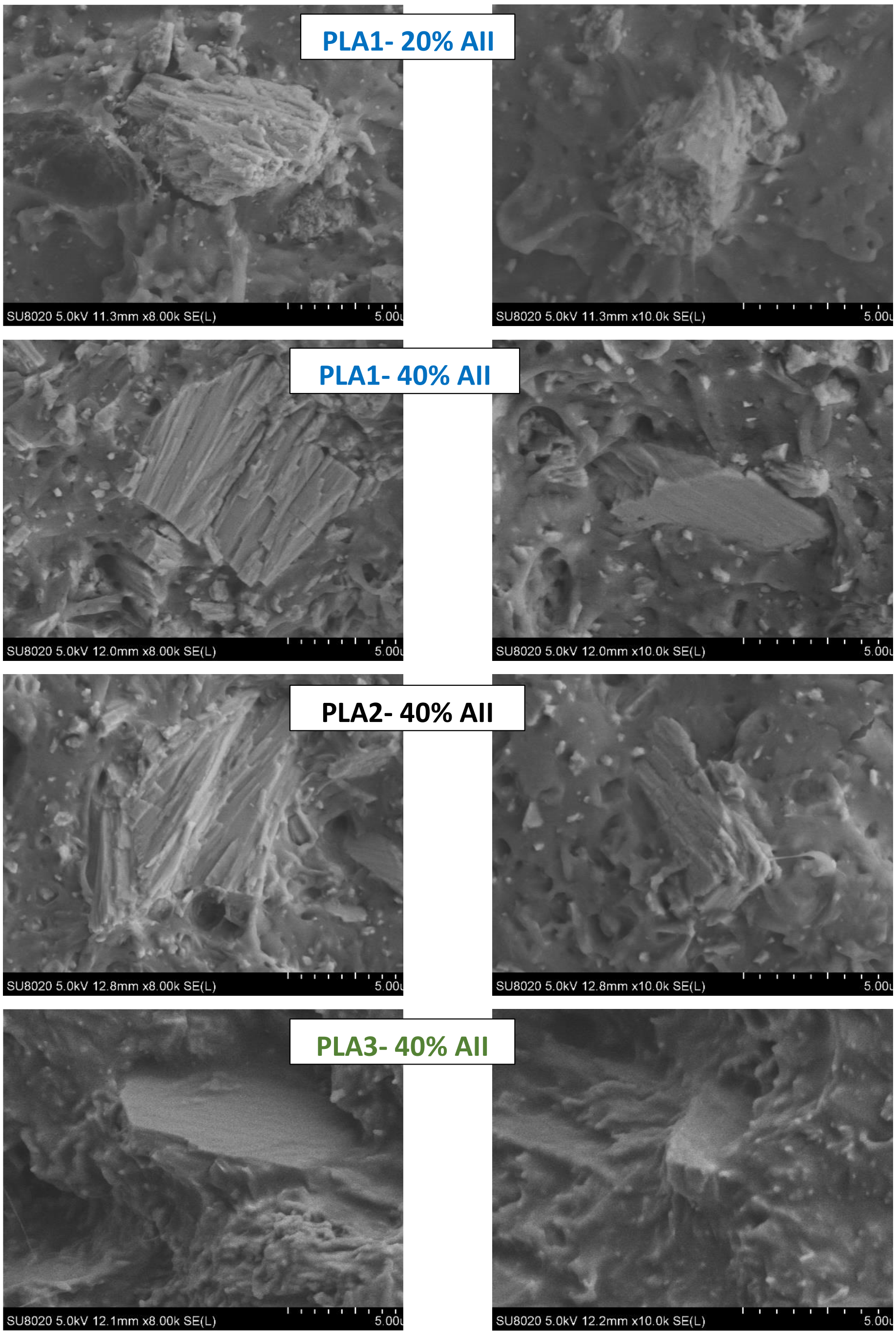

Figure S3. SEM images (SE mode) of PLA–AII composites performed on the fractured surfaces obtained after tensile testing.

Comment: *SEM images on the fractured surfaces obtained by tensile testing evidencing that at the interface PLA matrix–filler are present zones showing good interfacial adhesion(intimate physical contact) between components (which explain the good tensile properties), and regions of debonding, traditionally identified with a potential role in reducing the cracking and in dissipating the energy of impact solicitation, while increasing the impact resistance of mineral filled composites (NB: confirmation by impact testing only at 20% filler).*

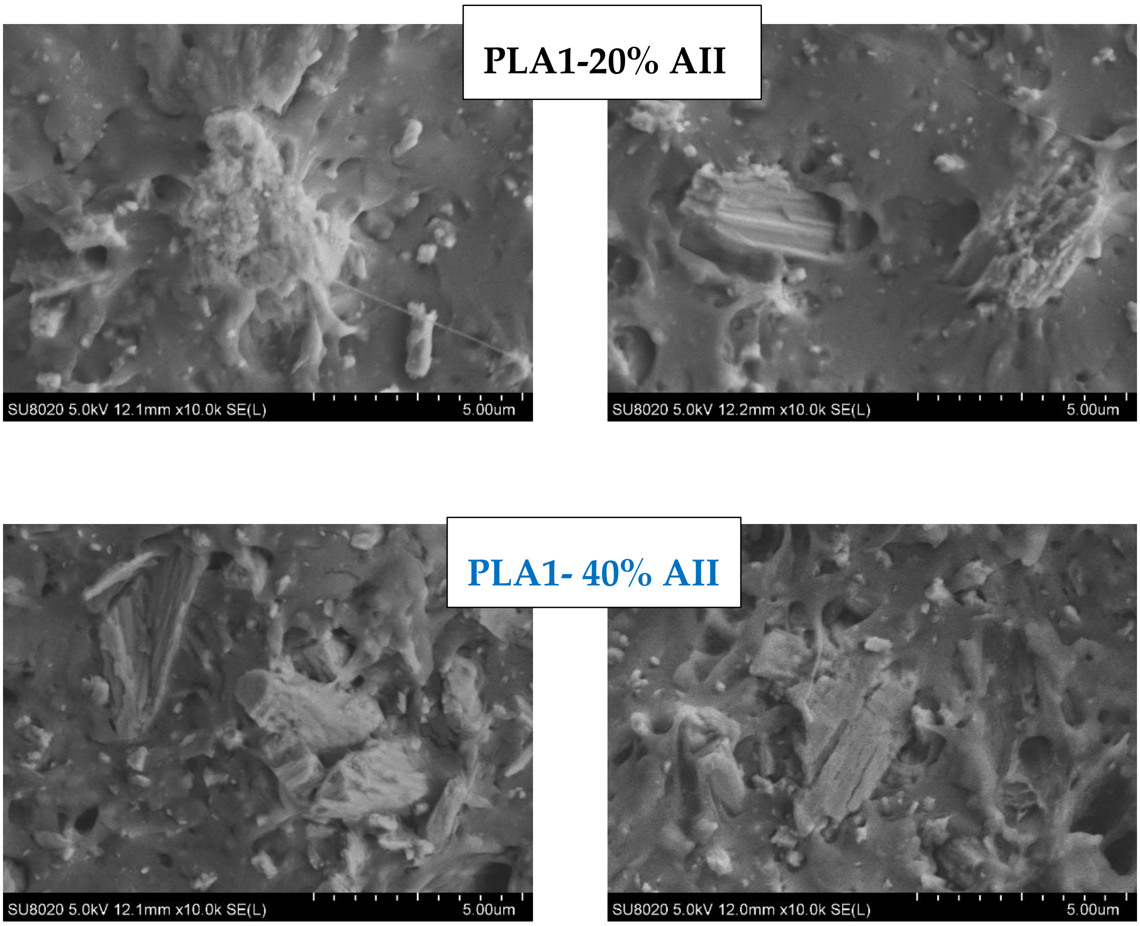

Figure S4. SEM images (SE mode) of PLA1–(20–40) % AII composites performed on fractured surfaces obtained after impact testing.

Comment: *First, it is noteworthy mentioning that during the tensile testing the PLA–AII composites have been stretched at low speed (1mm/min) until they break, whereas in impact tests a sudden shock is given to the material at high deformation rate, and this leads to the fracture of tested (notched) specimens. Once more the SEM pictures of PLA–AII composites are evidencing that at the interface matrix – filler are present regions assessing good adhesion between components (PLA–AII), and zones of debonding or shear yielding, with a potential role in reducing the cracking and in dissipating the energy of impact solicitation, while increasing the impact resistance (NB: behavior mostly evidenced at 20% filler).*

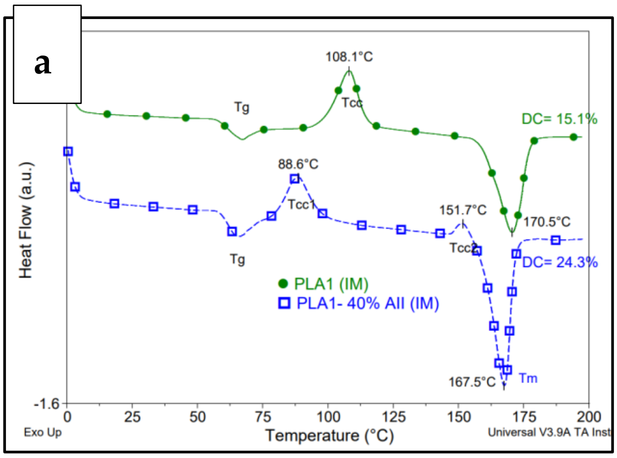

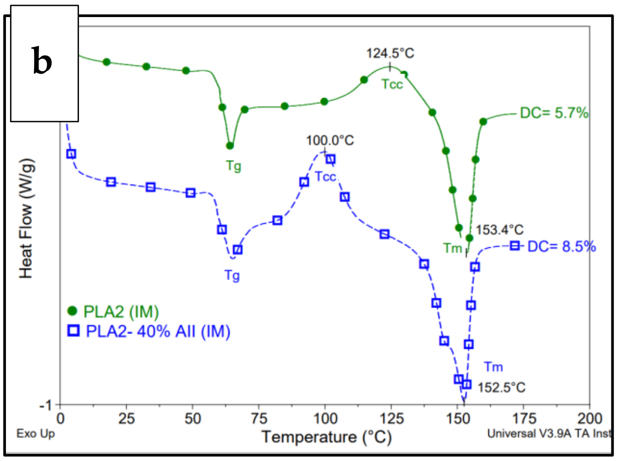

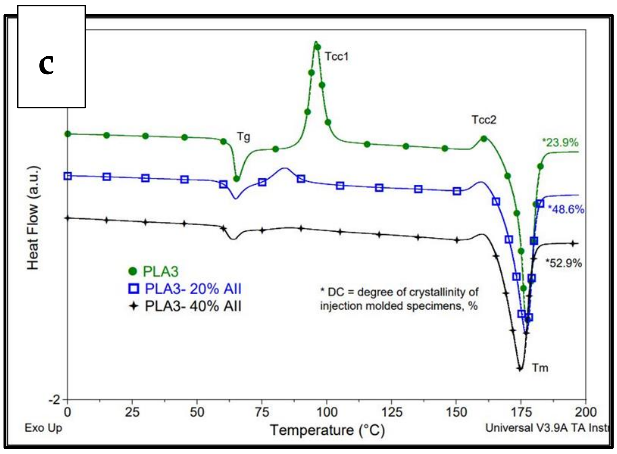

Figure S5 (a-c). Comparative DSC analyses on injection molded specimens of PLA and PLA–AII composites assessing for a higher degree of crystallinity (DC) of PLA3–AII composites. (First DSC scan, 10°C/min).

Comment: *The most remarkable enhancements of thermal properties obtained for PLA3–AII composites, i.e., an increase of Vicat softening temperature up to 160°C by comparison to about 62°C (the neat PLA3), was primarily ascribed to the high level of crystallinity.*

In fact, the IM tests highlight that the association of PLA3 with AII is leading to PLA composites characterized by remarkable crystallization kinetics, that allows the production of items characterized by high DC. Indeed, the comparative DSC analyses (Fig. S5c) performed on IM specimens, confirms their high DC (49-53%), results that are in good agreement with those presented in the section thermal characterizations (NB: by DSC) included in the paper.

On the other hand, PLA2 and PLA2–AII composites are remaining mostly in the amorphous state (DC lower than 10%) due to their low kinetics of crystallization (NB: PLA grade with high D-isomer content), whereas a significant increasing of crystallinity is seen in the case of PLA1–40% AII composites (DC above 24%, on specimens of 3 mm thickness obtained by IM).
